# Degradation of cofilin is regulated by Cbl, AIP4 and Syk resulting in increased migration of LMP2A positive nasopharyngeal carcinoma cells

**DOI:** 10.1038/s41598-017-09540-3

**Published:** 2017-08-21

**Authors:** Murat R. Gainullin, Ilya Yu Zhukov, Xiaoying Zhou, Yingxi Mo, Lidiia Astakhova, Ingemar Ernberg, Liudmila Matskova

**Affiliations:** 1 Department of Microbiology, Tumor and Cell Biology, Karolinska Institutet, Sweden; 2 Central Research Laboratory, Nizhniy Novgorod State Medical Academy, Nizhniy Novgorod, Minin Sq. 10/1, 603005 Russia; 3 Institute of Information Technology, Mathematics and Mechanics, Nizhniy Novgorod State University, Nizhniy Novgorod, Gagarin Av. 23, 603950 Russia; 4 Institute of Biology and Biomedicine, Nizhniy Novgorod State University, Nizhniy Novgorod, Gagarin Av. 23, 603950 Russia; 5 Medical Research Center, Guangxi Medical University, Nanning, China; 6 Department of Research, Affiliated Tumor Hospital of Guangxi Medical University, Nanning, China; 7 Institute of Food Science and Technology, Kemerovo, Russia

## Abstract

Expression of cofilin is directly associated with metastatic activity in many tumors. Here, we studied the role of Latent Membrane Protein 2 A (LMP2A) of Epstein-Barr Virus (EBV) in the accumulation of cofilin observed in nasopharyngeal cancer (NPC) tumor cells. We used LMP2A transformed NPC cell lines to analyze cofilin expression. We used mutation analysis, ectopic expression and down-regulation of Cbl, AIP4 and Syk in these cell lines to determine the effect of the LMP2A viral protein on cofilin degradation and its role in the assembly of a cofilin degrading protein complex. The LMP2A of EBV was found to interfer with cofilin degradation in NPC cells by accelerating the proteasomal degradation of Cbl and Syk. In line with this, we found significantly higher cofilin expression in NPC tumor samples as compared to the surrounding epithelial tissues. Cofilin, as an actin severing protein, influences cellular plasticity, and facilitates cellular movement in response to oncogenic stimuli. Thus, under relaxed cellular control, cofilin facilitates tumor cell movement and dissemination. Interference with its degradation may enhance the metastatic potential of NPC cells.

## Introduction

Close to 100% of non-keratinizing nasopharyngeal carcinomas (NPC) are associated with EBV^1^. The virus is a risk factor for NPC development, and most likely contributes to its tumorigenesis^2^. The virus resides in a latent state in tumor cells, with a restricted pattern of viral gene expression^3^. Latent Membrane protein 2 A (LMP2A) is commonly detected in EBV-positive NPC cells *in vivo* ^4^. It was shown *in vitro* that LMP2A promotes survival of pro-tumorigenic cells^5^ and imposes a migratory phenotype on epithelial cells^6, 7^. Previous studies have demonstrated that the Syk tyrosine kinase is targeted by LMP2A. LMP2A mediates constitutive Syk activation but also induces Syk degradation, resulting in a persistent low-level Syk activation^8^. LMP2A associates with Syk at an ITAM tyrosine motif and with the E3 ubiquitin ligase AIP4 at a tandem WW domain, both of which are located within the N-terminal 119 amino acid long intracellular domain^9^. It is also known that Syk binds and activates the Cbl E3 ubiquitin ligase^10^. Cbl ubiquitin ligases function as negative regulators of cell signaling^11^. AIP4 regulates Cbl function by binding and labeling it for degradation^12^ and its juxtaposition with Cbl in the LMP2A protein complex accelerates the turn-over of Cbl.

In order to further elucidate the mechanism by which LMP2A impacts on cellular homeostasis, we performed a large-scale search for novel LMP2A-binding proteins by mass-spectrometric analysis (MS). Using a chimeric construct, containing the C- terminal part of LMP2A, we identified cofilin as a binding partner.

Cofilin is an actin depolymerising factor (ADF). As a main component of the cytoskeleton, actin defines not only cellular shape, but also impacts on cellular homeostasis. Actin fibers at the cellular periplasm are dynamic structures. Rapid assembly and disassembly of the actin network is a prerequisite for cell migration in a wide variety of physiological and pathological processes, such as embryonic development, wound healing and tumor cell invasion. The proteins of the ADF/cofilin family are essential regulators of this actin dynamics^13^.

Cofilin is constitutively expressed but normally kept in an inactive form by several mechanisms. Cofilin is inactivated by phosphorylation at Ser3 by the LIMK1 serine/threonine kinase^14^. Impairment of the LIMK/cofilin pathway due to downregulation of p57kip² was reported in NPC cells, leading to cell invasion^15^.

Cofilin is kept inactive at the plasma membrane by binding to phospho-inositol 4,5-phosphate (PIP2)^16^.

Interestingly, also the inactive form of cofilin influences cellular behaviour. PIP2 bound cofilin activates phospholipaseD1 (PLD1), resulting in phosphatidic acid (PA) production, which was reported to facilitate Listeria monocytogenes invasion^17^. PA is reported to be important for adhesion and chemotaxis as well^10^.

A variety of post-translational modifications of cofilin were reported so far, including S-nitrosylation^18^, glutathionylation^19^, and oxidation on cysteines^20^. Cofilin undergoes modification with complex carbohydrates^21^, which enables cofilin to serve as a sensor for a multitude of extracellular signals including survival responses. Targeting cofilin was shown to suppress breast cancer metastasis via disruption of the cofilin-actin interaction^22^.

There are indications that cofilin turn-over is regulated by the proteasomal system^23–25^, however, the E3 ligase involved was not identified. In this study, we provide evidence that a direct interaction with proteins in the LMP2A-assembled signalling scaffold interferes with the proteasomal degradation of cofilin. In addition, our data suggest the involvement of the Syk tyrosine kinase in this process. The catalytic activity of Syk was reported to counteract activation of cofilin^26^. Our analysis of cofilin ubiquitination further suggests that cofilin is subject to ubiquitination by two E3 ubiquitin ligases, Cbl and AIP4, both components of the LMP2A signaling scaffold with different effects on cofilin stability and function. We test the impact of LMP2A on cofilin and cellular migration through perturbations of the proteasomal system.

## Results

### LMP2A binds cofilin and interferes with its proteasomal degradation

**Figure 1:**
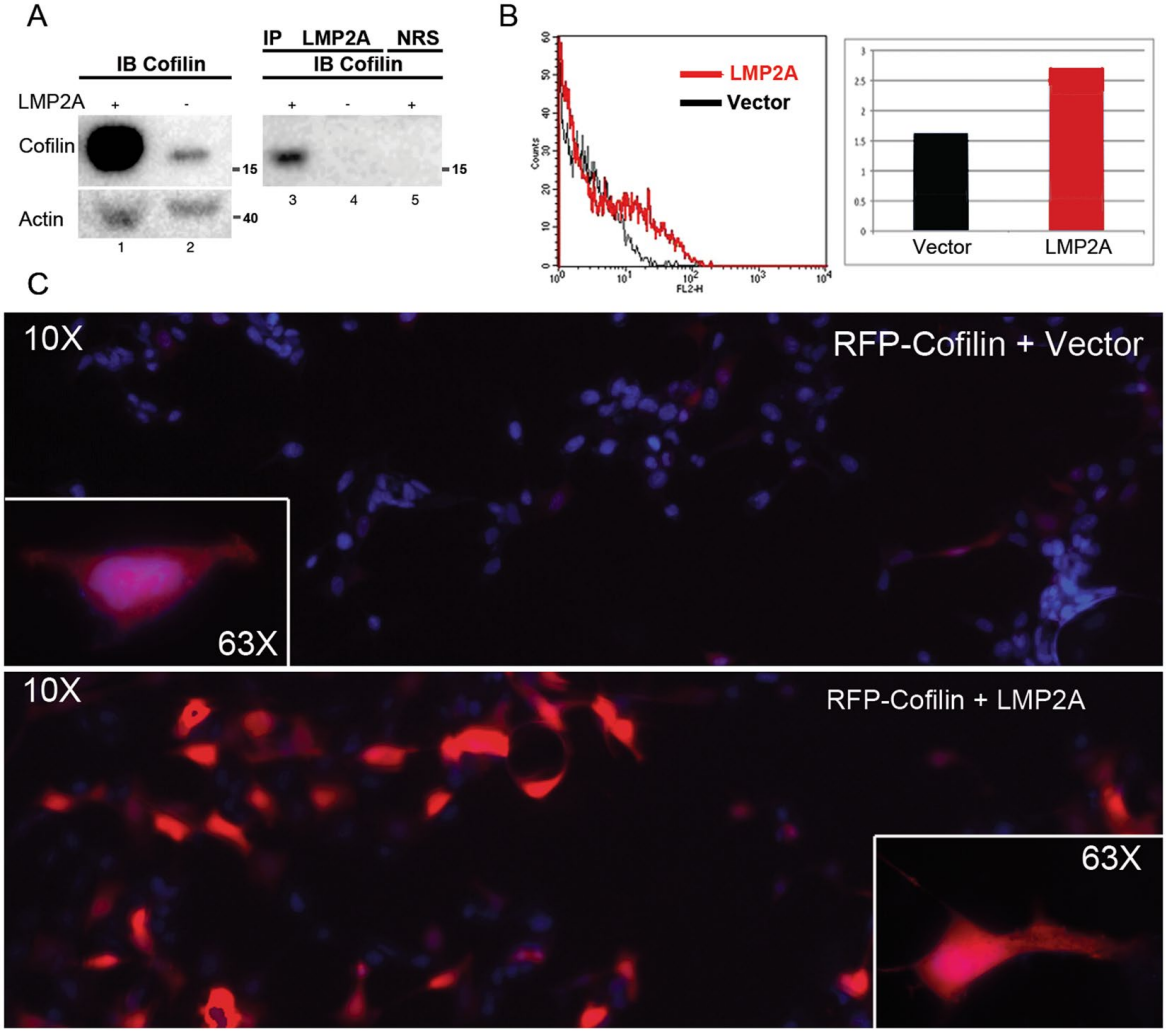
LMP2A binds cofilin and influences its stability. (**A**) WB analysis of cofilin levels in wcl of the NPC cell line CNE2 (lanes 1 and 2), lanes 3 and 4 show IP LMP2A, lane 5 shows IP NRS, lanes 3–5 probed with cofilin antibody. Full-length blot is presented in Supplementary Figure S5. (**B**) FACS analysis of cofilin expression in HEK293 epithelial cells. Histogram plot in the left panel shows red fluorescence intensity (FL2-H) on the horizontal axis and cell count on the vertical axis. Cells were transiently transfected with RFP-cofilin in combination with either an empty vector plasmid (black) or a LMP2A coding plasmid (red). Right panel: Staple diagram showing the geometric mean values of fluorescence intensities of RFP-cofilin in the FACS analysis shown in the left panel. (**C**) Microscopic analysis of HEK293 epithelial cells transiently transfected either with RFP-cofilin alone (upper panel) or together with LMP2A (lower panel), at 10x magnification. Insertions show single cells from the corresponding cultures at 63x magnification. Equal input of RFP-cofilin was used in both transfections.

In Fig. 1A, we show expression of cofilin in immunoblots of WCL from LMP2A positive (lane 1) and LMP2A negative cells (lane 2) probed with anti-cofilin antibody. Equal input of protein from LMP2A positive and negative cells is shown by the actin controls in the lower panel of Fig. 1A (lanes 1 and 2 respectively). It demonstrates that the steady state levels of cofilin are increased in LMP2A positive cells as compared to LMP2A negative cells. In lanes 3 and 4 we show that cofilin is detected in LMP2A immunoprecipitates from LMP2A expressing cells (lane 3) but not from LMP2A negative cells (lane 4) and is not detected in immunoprecipitates with an irrelevant antibody (lane 5). This provides confirmation that LMP2A binds cofilin. We also show that cofilin is up-regulated in LMP2A positive cells already at the gene expression level (Fig. S1).

The accumulation of cofilin in LMP2A positive cells was also confirmed by FACS analysis (panel B) and fluorescence imaging (panel C). Panel B in Fig. 1 shows FACS analysis results demonstrating that LMP2A expressing cells have an increased RFP-cofilin expression (red curve) as compared to LMP2A negative cells (black curve). The staple diagram on the left (Fig. 1 panel B) summarizes the quantitative difference in mean fluoresce values between the two cell populations.

Figure 1, panel C shows fluorescent images of HEK293 cells, cotransfected either with RFP-Cofilin and pCDNA vector (upper panel) or with RFP-cofilin and pCDNA 4xFLAG-LMP2Awt plasmid. The marked difference in fluorescence intensity of RFP-cofilin in the LMP2A expressing cells (Fig. 1C lower panel) as compared to the vector-cotransfected cells (Fig. 1C upper panel) agrees with the marked stabilization of cofilin that is observed in the presence of LMP2A, as shown in Fig. 1A and B.

**Figure 2:**
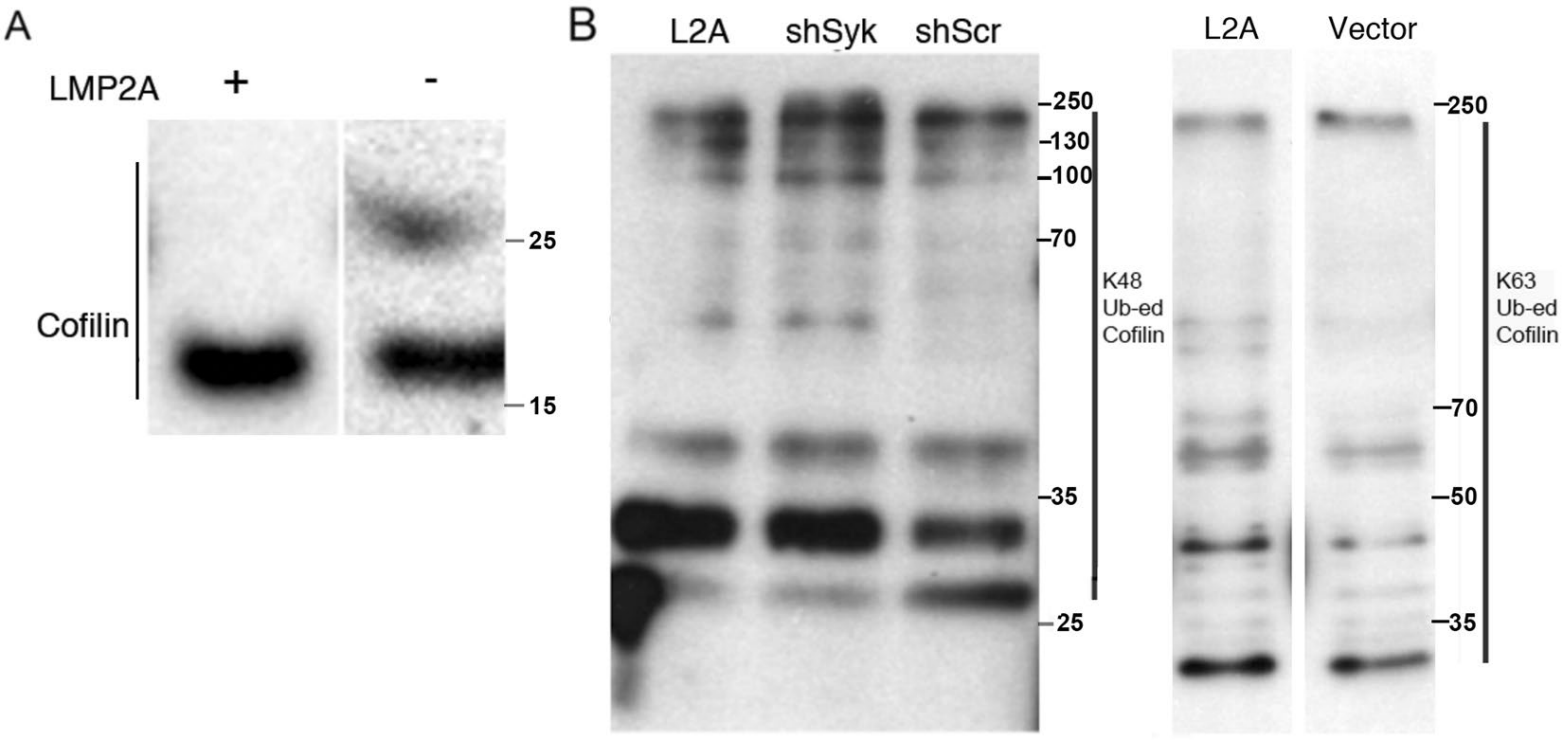
LMP2A expression interferes with ubiquitin modification of cofilin and with ubiquitin-branching patterns in cofilin immunoprecipitates. CNE2 NPC cells were treated with the MG132 proteasome inhibitor prior to lysis to prevent degradation of ubiquitinated proteins. (**A**) Whole cell lysates (wcl) of CNE2 cells were immunoblotted with cofilin antibody. Ubiquitin modifications of the proteins in the cofilin immunoprecipitates were probed with either K48-ubiquitin specific antibody (**B**) or K63-ubiquitin specific antibody (**C**). Full-length blots are presented in Supplementary Figure S6.

Since overexpression of cofilin was observed in LMP2A expressing cells (Fig. 1), we asked whether the known interaction of LMP2A with two ubiquitin ligases, AIP4 and Cbl might alter the stability of cofilin in LMP2A expressing cells. We first observed that cofilin, in LMP2A negative CNE2 cells, treated with the proteasome inhibitor MG132, underwent a modification that increased its MW to approximately 27 kDa (Fig. 2, panel A, right lane). LMP2A expressing CNE2 cells, however, lacked this additional 27 kDa band (Fig. 2, panel A, left lane). This indicated that cofilin, in LMP2A negative cells, underwent a modification that increased its MW by about 8 kDa. We asked whether cofilin might be modified differently by ubiquitin in LMP2A expressing and control cells. To this end, we undertook to analyze the patterns of K48 linked and K63 linked ubiquitin complexes in cofilin immunoprecipitates from CNE2 cells after inhibition of proteasome activity with MG132.

Inhibition of proteasomal degradation induces accumulation of additional, higher order molecular forms, which are recognized by K48 and K63 specific ubiquitin antibody (Fig. 2 panels B and C respectively). As expected, the patterns of K48 and K63 branched ubiquitin chains in the cofilin immunoprecipitates are significantly different. There are also quantitative differences between the cofilin immunoprecipitates from LMP2A positive and negative epithelial cells.

When probed with K48 specific ubiquitin antibody, the LMP2A positive and shSyk transfected cells (Fig. 2, panel B, lanes labelled LMP2A and shSyk) showed a reduced amount of K48-linked mono Ub-conjugates of cofilin, in particular with respect to the 27 kDa band (Fig. 2B), as compared to control cells treated with scrambled shRNA (Fig. 2B, panel B, lane shScr). Conversely, there was an increase in (poly-) ubiquitinated cofilin species in the LMP2A positive NPC cells as recognized by the K63 specific ubiquitin antibody (Fig. 2C).

### Two E3 ubiquitin ligases control cofilin expression

**Figure 3:**
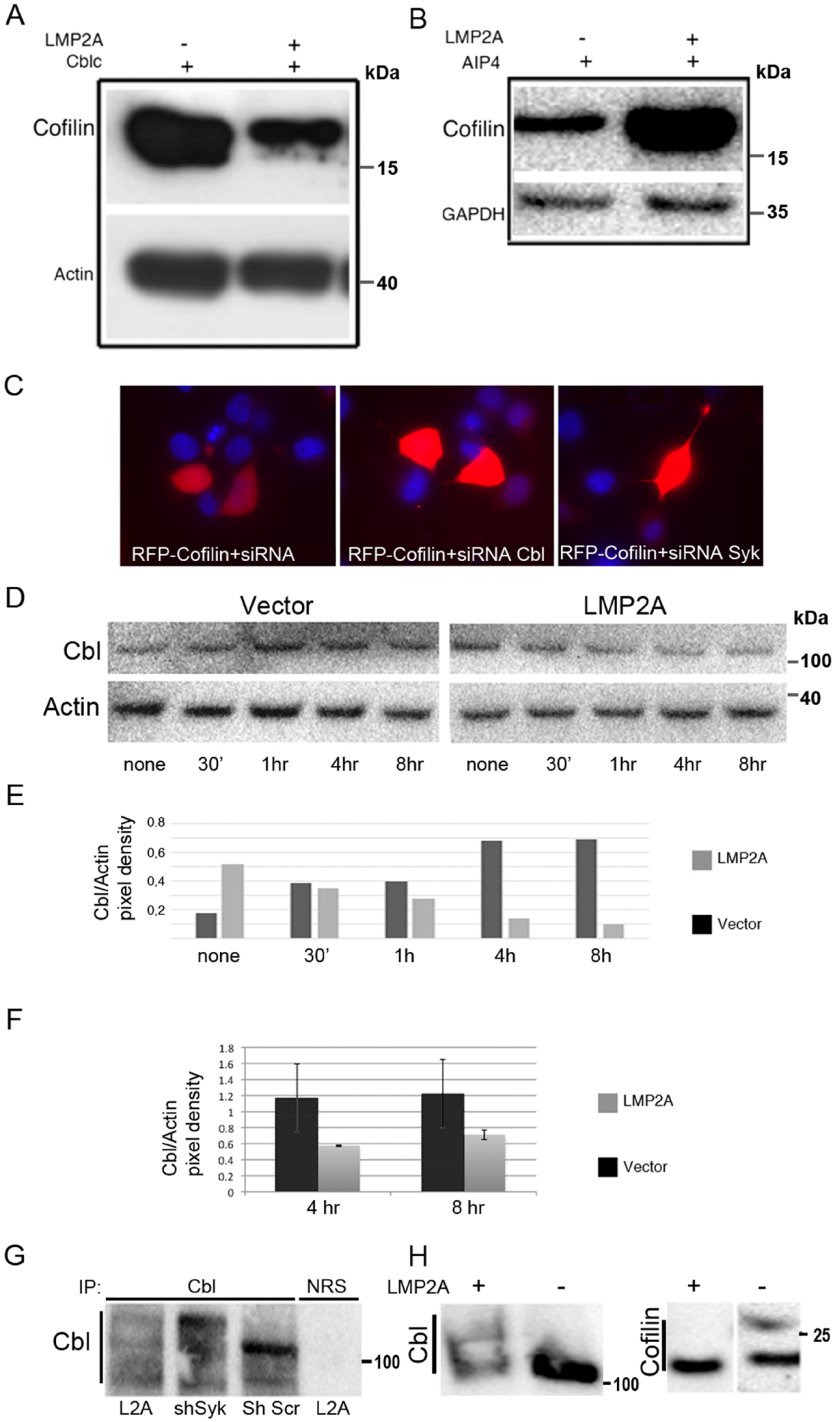
Two ubiquitin E3 ligases are involved in the regulation of cofilin protein stability. WB analysis of cofilin expression in HEK293 cells upon transient expression of either wt Cbl (panel A) or wt AIP4 (panel B) in the presence of LMP2A (left lanes), or in its absence (right lanes). Full-length blots are presented in Supplementary Figure S7A,B. Panel C shows microscopic images of RFP-Cofilin expression in HEK 293 cells, either co-expressed with an irrelevant siRNA (panel C, left image), with siRNACbl (middle image) or with siRNASyk (right image). (**D**) WB analysis of Cbl expression in vector transfected or LMP2A expressing HEK293 cells upon inhibition of proteasomal degradation with MG132. Panel E: Quantitation of Cbl expression from panel D. The staple diagram in panel E shows the ratio of Cbl over actin at the indicated time points after addition of MG132 to the vector (black bars) or LMP2A expressing (gray bars) HEK 293 cell cultures. The staple diagram was generated using the Image J program. Panel F shows the ratio of Cbl over actin in stably transformed LMP2A positive (grey bars) or negative parental (black bars) CNE1 NPC cells. The immunoprecipitations in panel G show the appearance of post-translationally modified (larger) forms of Cbl in LMP2A or shRNAsyk expressing cells. Anti-Cbl and anti-cofilin immunoblots in Panel H show that additional larger forms of Cbl appear (left panel) while one larger form of cofilin (27 kDa) disappears (right panel) in LMP2A positive TWO3 cells. Full-length blots are presented in Supplementary Figure S7C–E.

To further understand the regulation of cofilin protein stability, we investigated the involvement of two E3 ligases, AIP4 and Cbl, and the Syk tyrosine kinase, all of which are components of the LMP2A protein complex. We found that exogenous expression of wt Cbl resulted in decreased cofilin levels in LMP2A expressing CNE1 cells (Fig. 3A), while exogenous expression of AIP4 led to stabilization of cofilin at the protein level (Fig. 3B). Analysis of cofilin peptides after tryptic digestion (Fig. S2) also support the involvement of these two ubiquitin ligases.

In Fig. 3C, we show, by fluorescence imaging of individual cells, that siRNA-mediated down-regulation of Cbl (middle panel) or Syk (left panel) results in increased levels of RFP-cofilin as compared to cells treated with irrelevant siRNA (right panel). These siRNA experiments in LMP2A negative HEK293 cells show that cofilin is stabilized by siRNA-mediated downregulation of either Cbl (middle image) or Syk (right image) but not by a scrambled control siRNA (left image). This reproduces the stabilizing effect on cofilin effected by LMP2A-mediated downregulation of Cbl and Syk. This effect is also observed by western blot (Fig. S3A) and by FACS analysis (Fig. S3B). The rapid disappearance of Cbl upon inhibition of proteasomal degradation in LMP2A positive cells (Fig. 3D, left panel) as compared to vector transfected cells (Fig. 3D, right panel) suggests that the increased turnover of Cbl in LMP2A cells depends on a different protein degradation system. Panel E shows quantitation of the pixel density ratio of Cbl over actin in the WB shown in panel D using the Image J program, showing that while Cbl levels were fourfold increased over 8 hours in LMP2A negative cells in the presence of the proteasome inhibitor MG132, there was a fivefold reduction in Cbl levels in LMP2A expressing cells. Panel F shows the reduction in the ratio of Cbl over actin expression in LMP2A expressing (grey bars) as compared to vector transfected CNE1 NPC cells.

In Fig. 3, panel G we show that IP with anti-Cbl antibodies also detects an additional, larger form of Cbl in stably LMP2A expressing CNE1-cells (lane 1) as well as in CNE1-cells stably expressing an shSyk shRNA from an integrated retroviral construct (lane 2) but not in CNE1 cells stably expressing a scrambled control shRNA (Fig. 3, panel G, lane 3) where only the normal (100 kDa) size of Cbl is detected. This suggests that Cbl undergoes posttranslational modifications specific for LMP2A expressing cells, or else where Syk expression (and thus also Cbl activation) is suppressed. Of note, it was previously reported^12^ that AIP4 targets Cbl for lysosomal degradation. Consequently, the stabilization of cofilin levels observed by us, is likely due to the reduced Cbl levels in LMP2A expressing cells. Thus, the disappearance of the 27 kDa form of cofilin in LMP2A expressing TWO3 NPC cells (Fig. 3H, right panel), correlates with a reduced expression of Cbl, shown in the left-hand panel of Fig. 3H. This likely reflects the previously established effect of LMP2A expression on the stability of Syk^9^. Cbl was also reported as a target for proteasomal degradation^27^. Thus, the two E3 ligases, Cbl and AIP4, have opposite effects on cofilin expression. While wt Cbl negatively affects cofilin expression, expression of wt AIP4 in HEK293 cells, on the contrary, correlates with increased cofilin expression (Fig. 3 panels A and B). These studies confirm that Cbl plays an important role in the regulation of cofilin stability.

### LMP2A exempts cofilin from degradation to mediate increased cellular motility

**Figure 4:**
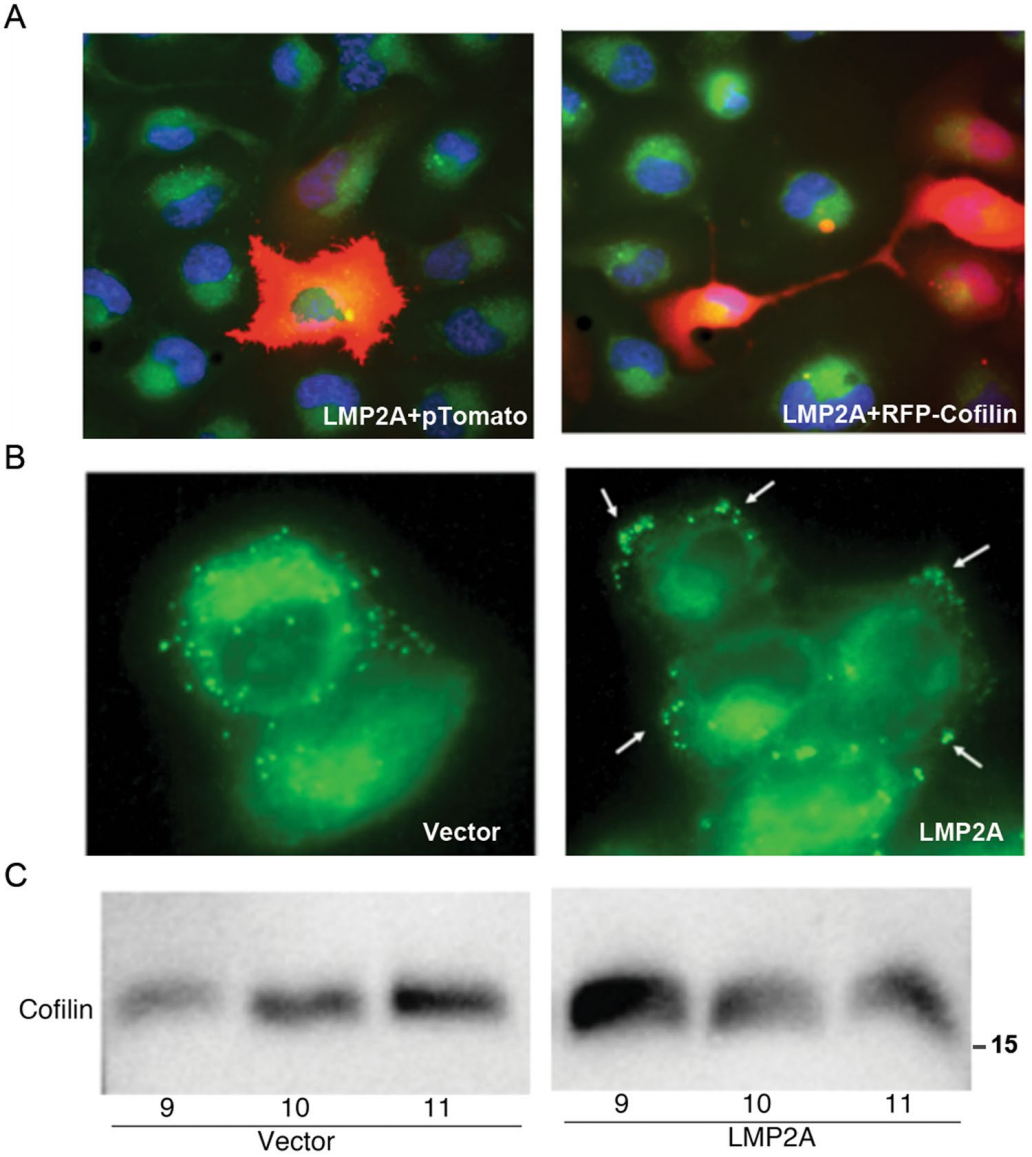
LMP2A facilitates cofilin accumulation at the cellular periphery. Fluorescence analysis of cofilin expression. Panel A CNE2 cells transiently transfected with LMP2A, together with a plasmid coding for the fluorescent protein pTomato (left panel). CNE2 cells transiently transfected with LMP2A, together with a plasmid, coding for RFP-cofilin (right panel). (**B**) LMP2A induced accumulation of lipid droplets in CNE2 NPC cells. Specific distribution at the periphery of the cells is indicated with arrows. Fluorescence analysis with the neutral lipid specific fluorescent dye Bodipy 495/503 in cells transfected with vector control (left panel) or with an LMP2A construct (right panel). (**C**) WB analysis of cofilin distribution after ultracentrifugation in a discontinuous Optiprep gradient. Fractions are numbered from the top.

To study whether the LMP2A-mediated stabilization of cofilin turn-over affects cofilin function, we investigated the cellular localization of cofilin. HEK293 cells were transfected with a LMP2A coding plasmid in combination with either a red fluorescent control vector (pTomato) or a RFP-cofilin encoding plasmid. Transfected cells were detected with red fluorescence (568 nm wavelength). Coexpression of LMP2A and cofilin induced morphological changes (Fig. 4A right panel), which were absent in the LMP2A and pTomato transfected cells (Fig. 4A the left panel). In LMP2A-expressing cells, cofilin was redistributed towards peripheral cellular structures characteristic for moving cells.

To show that LMP2A, stably LMP2A expressing (Fig. 4B, right panel) or control CNE2 cells (Fig. 4B, left panel) were fixed and stained with Bodipy, a neutral lipid specific fluorescent dye (468 wavelength). Similarly to the peripheral subcellular distribution of cofilin (Fig. 4A, right panel), and to the early demonstration that LMP2A is expressed in clusters in the membrane^28^, lipids droplets were also localized in clusters at the cellular periphery in the LMP2A transfected cells as compared to the uniform distribution in the control cells. In line with this, we observed that cofilin floated closer to the top of discontinuous Optiprep gradients in LMP2A positive cells, while in LMP2A negative cells, cofilin remained closer to the bottom of the gradient (Fig. 4C).

The effect of LMP2A and cofilin on cellular migration was measured by a scratch wound assay in stably LMP2A transfected or control CNE2 cells, which were transiently transfected with RFP-cofilin. At 24 hrs post-transfection a scratch wound was introduced.

**Figure 5:**
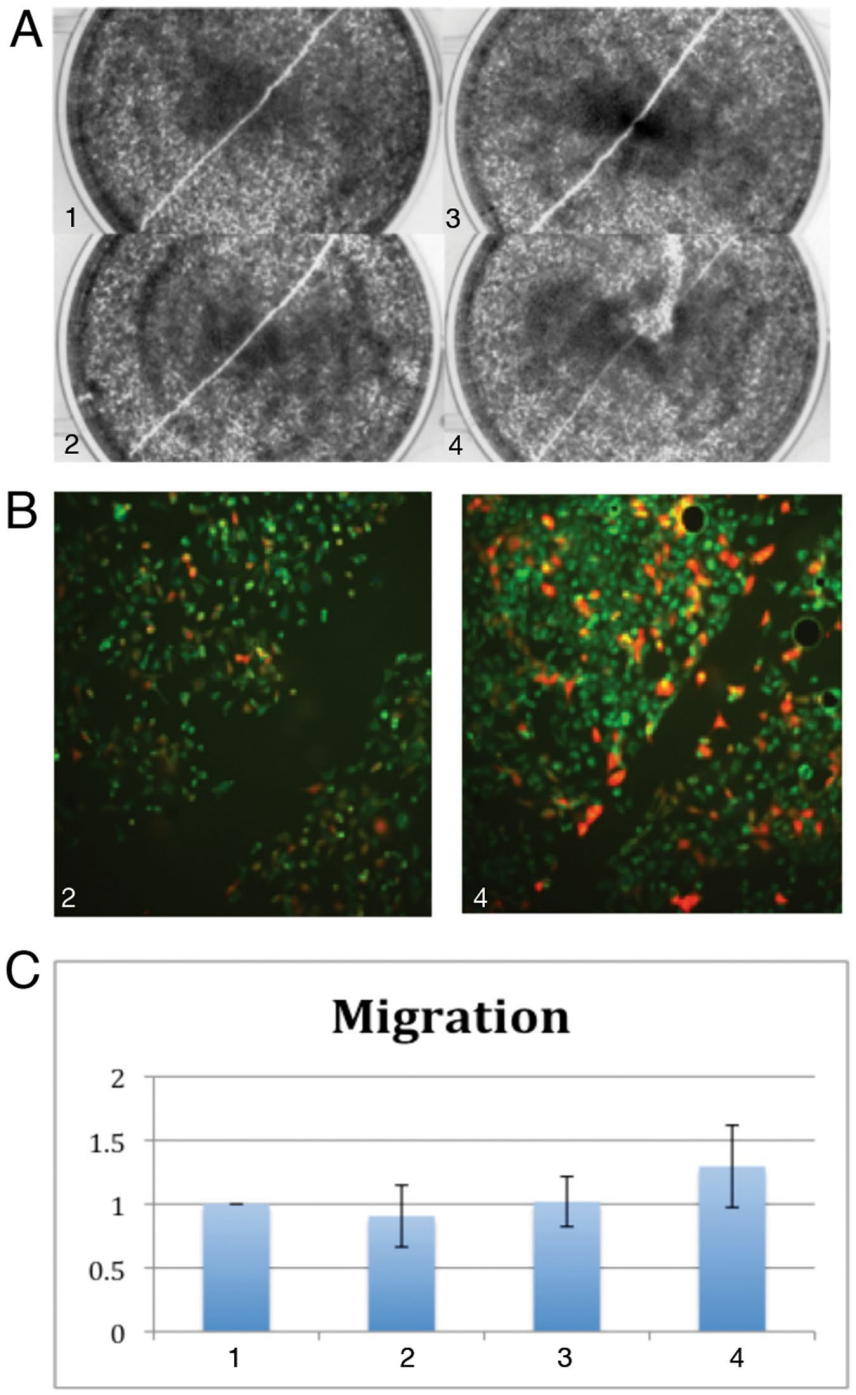
LMP2A facilitates cofilin mediated migration of NPC cells. Scratch assay. (**A**) Phase-contrast images of LMP2A positive cells transiently transfected with a vector plasmid before introducing a scratch (plate 1) and 18 hr later (plate 2), and together with a plasmid encoding RFP-cofilin (plates 3 and 4 respectively). (**B**) Epifluorescent microscope images of the scratches introduced 18 hr previously in LMP2A negative and positive cells. (**C**) Graph shows quantitation of the differences in migration of cells on plates 1–4 in panel A (Y axis values correlate with rate of cell migration, which is the inverse value of the gap closures in different cells).

LMP2A-positive cells, transfected with cofilin (Fig. 5A, plates 3 and 4) migrated fastest, resulting in a rapidly closing gap as compared to similarly treated cofilin transfected CNE2 control cells (Fig. 5A, plates 1 and 2). The red fluorescence in RFP-cofilin expressing cells was also more intense in the LMP2A background (Fig. 5B, right panel) than in CNE2 control cells (Fig. 5B, left panel), in line with our finding that LMP2A expression stabilizes cofilin in HEK293 cells (Fig. 1). The LMP2A and RFP-cofilin positive cells were both larger in size and accumulated at the border of the gap to a higher degree than did the control cells (Fig. 5B, right panel). Figure 5C presents a quantitative measurement of gap closure as demonstrated on plates 1 to 4 of Fig. 5A. Gap distances were captured with the DpxView Pro software at 15 points along the scratch. Staples 1 and 3 represent averages of initial gap distance values normalized to 1, with SD. Staples 2 and 4 represent the ratio of initial value over 24 h gap distance value, to reflect migration rate of cells.

### Cofilin is overexpressed in NPC tissue samples

**Figure 6:**
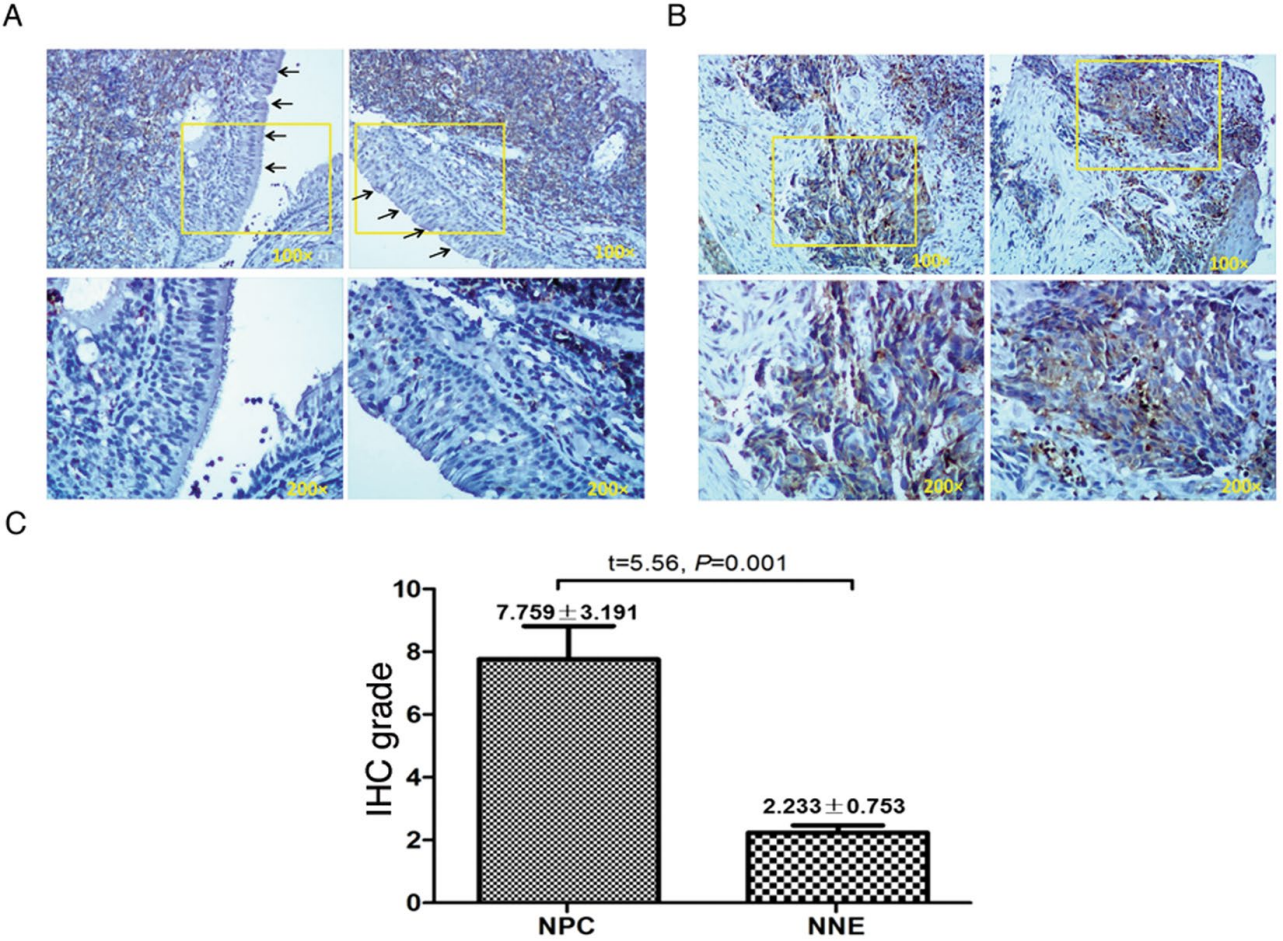
Nasopharyngeal carcinoma (NPC) samples express significantly more cofilin than normal nasopharyngeal epithelium (NNE). Representative IHC slides of cofilin stained NNE (**A**) and NPC (**B**) samples are shown. Black arrows denote NPC tissue. Panel C Staple diagram shows the statistical analysis of cofilin expression in NPC and NNE tissues.

We analysed cofilin protein expression *in vivo*, in NPC tumors (Panel B) and in NNE tissue samples (Panel A) after IHC staining. In most of the NPC biopsy samples a large proportion of cancer nests (Panel B, enlarged sections at 200x) were cofilin positive. IHC grade of cofilin was significantly higher in NPC cancer nests (7.759 ± 3.191) than NNE tissues (2.233 ± 0.753, P = 0.001), Staple diagrams in panel C show a summary of IHC grade value for the tissue samples evaluated, based on the scoring of IHC peroxidase staining in an average of 5 fields/tissue section (Fig. 6).

## Discussion

We demonstrate that the viral LMP2A protein associates with cofilin, resulting in cofilin stabilization (Fig. 1). We hypothesize that this effect of the viral protein on cofilin half-life facilitates cellular motility. In this article we explore the mechanism of the LMP2A-mediated stabilization of cofilin.

Cofilin was recently reported to undergo proteasomal degradation^23–25^, although a cofilin specific E3 ubiquitin ligase was not identified. Here we confirm the conventional K48 ubiquitin modification of cofilin, and report the finding of the novel K63 ubiquitin chain extension for cofilin, which suggests that two E3 ubiquitin ligases are involved in ubiquitin modifications of cofilin.

We suggest that the E3 ubiquitin ligase Cbl catalyzes addition of a conventional K48-linked ubiquitin chain to cofilin. The Cbl level in LMP2A positive cells is reduced as a result of the assembly, by the viral protein, of a specific protein complex consisting of the two ubiquitin ligases Cbl and AIP4 and the tyrosine kinase Syk^8^, where Cbl is targeted for degradation.

The E3 ubiquitin ligases Cbl and AIP4 are known to jointly orchestrate the final steps in the process of cell surface receptor internalization^29^. During this process, AIP4 ubiquitinates Cbl, providing the targeting signal for its degradation^27^. The process is initiated by tyrosine phosphorylation of Cbl^10, 30, 31^. Tyrosine kinase activity, in turn, is also an inducible event^10^. While at a steady-state, kinases may separate the two ubiquitin ligases, protecting them from untimely degradation. We speculate that the Syk tyrosine kinase, was early reported to phosphorylate Cbl^32^ and found in a complex with AIP4^33^, functions as such a spacer. Our observations of higher molecular weight isoforms of Cbl in western blot analyses of cell lysates from cell lines where Syk is down-regulated or absent (Fig. 3G), are suggestive of its ubiquitination. In LMP2A positive cells, Syk is constitutively but weakly activated and simultaneously degraded^8^ which may contribute to an elevated rate of Cbl degradation leading to a predominance of AIP4 over Cbl activity^34^. AIP4 is known to specifically catalyze K63 ubiquitin conjugation^35^. Indeed, we detect unique species of lysine K63 Ub labelled cofilin in LMP2A positive or shSyk treated cells (Fig. 2C). The K63 ubiquitin-branched modification was previously reported to modulate signaling complexes assembled on the targeted proteins^36^. This type of ubiquitination was not previously described for cofilin. The K48 linked, mono-ubiquitinated 27 kDa form of cofilin, which is also detected at a low level in LMP2A positive and shSyk expressing cells Fig. 2B, can be attributed to residual Cbl expression in these cells. Our data on differential sensitivity of cofilin immunoprecipitates from LMP2A negative and positive CNE2 NPC cells to deubiquitinating enzymes (DUBs) are in line with this (Fig. S4). Cofilin immunoprecipitates from LMP2A cells are more sensitive to AMSH, the K63 specific DUB, while cofilin immunoprecipitates from LMP2A negative cells are more sensitive to Cezanne, the K11 specific DUB as judged by appearance of mono-, di-, tri- and tetra-ubiquitin species. Recently, K11 ubiquitination was associated with protein degradation^37^ while the K63 ubiquitination pattern serves to modify cellular signaling^36^.

We suggest that the two ubiquitin ligases, Cbl and AIP4, modify cofilin in different manners. Cofilin ubiquitylation catalyzed by Cbl is a prerequisite for cofilin degradation by the ubiquitin-proteasome system (UPS), whereas the AIP4 mediates addition of K63-linked multi- or oligo-ubiquitin chains. The latter type of ubiquitylation, is associated with activation of various pathways, e.g. cofilin targeting into the MVB organelle, as was shown for ERBB4^29^, or sorting to the lysosomes as in the case EGFR^38, 39^, i.e. processes that are involved in different aspects of protein metabolism and transport and proceed at different rates than proteasomal degradation.

In addition, the reduced expression and activity of Syk in LMP2A expressing cells may contribute to cofilin activation as it was reported that Syk negatively affects cofilin activity^26^.

Beyond mediating an increase in the levels of cofilin, LMP2A expression may influence its localization in the cells, due to the excessive accumulation of lipids that we observe at the periphery of LMP2A positive cells (Fig. 4B). Lipids may physically sequester cofilin at the protruding ends. This suggests that cofilin may acquire modifications in LMP2A expressing cells that favor its reversible association with lipid domains. Based on available tools for prediction of lipid modifications, cysteines 39 and 147 may be targets for palmitoylation^40^. Interestingly, it was reported that the periplasmic fraction of cofilin is active in redirecting apoptotic signaling into migratory^41, 42^. Thus, the effects of LMP2A on lipid accumulation and relocation of cofilin to lipid domains are in line with our observation of a more migratory phenotype in migration (scratch) assays (Fig. 5A).

Apart from the LMP2A-mediated interference with proteasomal degradation of cofilin, its interaction with the LMP2A protein complex may also interfere with other cofilin inactivating processes such as binding to PIP2 and phosphorylation by LIMK1 kinase^14, 16^.

Thus, in addition to mediating an increase in steady state levels of cofilin protein, association with LMP2A may also contribute to maintaining cofilin in an active state. Interference with cofilin and actin organization may be a mechanism behind increased cell surface expression in LMP2A positive cells of a number of receptors like CXCR4, VEGFR, EGFR (our preliminary data and^42^. Most probably, cofilin affects the delivery of cell surface receptors through the endocytic vesicle fusion machinery, as is the case for cofilin-assisted expression of GLUT4^43^. In the presence of growth factors, chemokines or other stimulatory signals, LMP2A-mediated regulation of cofilin expression can facilitate directional cellular movement, due to increased actin turnover.

The stabilization of cofilin by the LMP2A complex may affect immune responses. Cell surface expression of cofilin was reported recently as an autoantigen during apoptosis, capable of inducing the production of autoantibody^44, 45^.

In summary, we show that the LMP2A protein complex confers stabilizing K63-branched ubiquitin chains on cofilin through the action of AIP4, resulting in increased steady-state levels of cofilin. This effect is mediated by Syk catalytic activity which induces AIP4 dependent Cbl degradation. Targeting the cellular ubiquitination machinery is one way by which EBV, like many other viruses, interferes with cellular functions as part of their survival strategies^46^. Analysis of cofilin expression in histological sections of normal nasopharyngeal or NPC tissue (Fig. 6) supports our conclusion that cofilin expression is dysregulated in NPC tumor tissue. We propose that cofilin dysregulation may serve as a tumor hallmark of NPC, the cellular mechanism for which is suggested by the involvement of the viral LMP2A protein in dysregulation of ubiquitin-mediated cofilin turnover in the tumor cells.

Cells with increased cofilin expression have a propensity to form more cellular protrusions, contributing to increased cellular motility, phagocytosis and responses to growth factors.

Our finding on the LMP2A mediated inhibition of cofilin degradation warrants further investigation of the ubiquitylation pattern of cofilin and its functional relevance.

## Materials and Methods

### Regulatory and safety statements

All experimental protocols and procedures were performed in accordance with the regulations issued by the Swedish Environment Authority and the Environmental Authority of the Peoples Republic of China regarding recombinant DNA and microbiological work with genetically modified organisms (BSL1 and 2, where applicable). All work was carried out using experimental protocols approved by the respective Biosafety Committees of Karolinska Institutet and Guangxi Medical University, respectively, performed in laboratory facilities designed for this purpose. Work performed at the Institute of Biology and Biomedicine in Russia consisted of biophysical analyses of biologically inactivated protein preparations prepared at Karolinska institute for these analyses. Coauthors with other addresses performed their work while at the Karolinska Institute. All human materials were obtained with informed consent from the donors (see below).

### Ethics Statement

Ethical permission for this study was approved by the Research Ethics Committee of the First Affiliated Hospital of Guangxi Medical University (Nanning, China) and by the Regional Ethics Committee of Karolinska Institutet, Stockholm, Sweden, No. 00-302.

### NPC cell lines, primary tumors and NNE

Human embryo kidney (HEK)293 and NPC derived cell lines, CNE1, CNE2 stably transformed with shRNA Scrambled, shSyk, vector plasmid or LMP2A as described in ref. 6, TWO3, HONE1 were routinely cultivated in IMDM medium (Invitrogen, Carlsbad, CA, US), containing 10% fetal bovine serum (HyClone, UK Ltd, Northumberland, UK).

In total, NPC tissues from 9 patients (42.11 ± 10.06 years old, 4 males, 5 females) and epithelial tissues from 10 patients (52.3 ± 12.63 years old, 5 males, 5 females) with chronic nasopharyngitis, considered as controls, were obtained at the Department of Otolaryngology Head & Neck Surgery, First Affiliated Hospital of Guangxi Medical University (Nanning, China). Written informed consent was obtained individually from each participant in the study. Diagnoses were made by experienced pathologists according to the World Health Organization (WHO) classification. All NPC samples were nonkeratinizing carcinomas. All the tissue samples were collected before radiotherapy. Tissue blocks were formalin-fixed and paraffin-embedded (FFPE). Standard immunohistochemical methods were used to examine the distribution of cofilin in NPC tissues and normal controls as described previously^47^. Immunohistochemical (IHC) grades were assigned based on intensity and frequency of staining results and was performed by two independent investigators. Staining intensity was scored as negative (0), weak (+1), moderate (+2), or strong (+3). The frequency of positive cells in specific areas was scored as negative (0), less than 25% (+1), 25–50% (+2), 51–75% (+3), or more than 75% (+4). IHC grades were assigned by multiplying the intensity score by the frequency score as described previously^48^. The experimental protocols dealing with human samples were approved by the Research Ethics Committee of the First Affiliated Hospital of Guangxi Medical University (Nanning, China).

### cDNA constructs, transfections and cell treatment

pmRFP-N1 human cofilin wt plasmid was purchased from Addgene (50856).

The pCDNA 4xFlag-LMP2A wt construct was designed and kindly provided by Dr Rob Ingham, Edmonton, Canada and described in ref. 9. The wt AIP4 construct is described in ref. 9. The Cbl and AIP4 constructs were kindly provided by Dr. Gerry Gish, Toronto. The authenticity of all the constructs was confirmed by DNA sequencing.

The total amount of plasmids was kept constant throughout all transfections and corrected with empty vector plasmid if needed. Pre-designed siRNAs, siCbl ID 133804, siSyk ID 138814 and an irrelevant siRNA ID 4390843 (Ambion, Life Technology), were transfected at 100 pmol/well. and analyzed after 24 hr. The Lipofectamine2000 Transfection Reagent was used for all plasmid and siRNA transfections (cat No11668027, Thermo Fisher Scientific).

Treatment with the proteasomal inhibitor MG132 was performed as described in ref. 9.

### Optiprep gradient fractionation

Cell lysates were mixed with Optiprep reagent (AXIS-Shield PoC AS, Oslo, Norway) and loaded on a discontinuous density gradient as described previously for floatation analysis of membrane proteins^49^. Centrifugation was performed at 25 000 rpm for 16hr at 4 C. One milliliter fractions were harvested from the top. Proteins were concentrated by Trichloroacetic acid (TCA) precipitation. The TCA traces were washed away with acetone, the fractions were dried and dissolved in Western blot sample buffer and analyzed by Dotblot.

### Flow cytometry and confocal microscopy analysis

To analyze the expression of cofilin, adhesive cells were trypsinized, washed with PBS twice and resuspended in PBS. Fluorescence was measured and analyzed on a flow cytometer (FACS Calibur, Becton Dickinson, San Jose, CA, US) with CellQuest software. Mean fluorescent intensities in gated fractions of cells was compared.

A total number of 50 000 cells were seeded in 6 wells and allowed to attach overnight. Adherent cells were fixed with 4% formaldehyde for 15 minutes, permeabilized with 0.5% Triton X-100 for 10 minutes. Cell nuclei were counterstained with DAPI. Images were acquired using a Leica DMRE microscope with HiPic software (Leica, Bensheim, Germany).

Lipid droplet specific fluorescent dye BODIPY (D3922, Molecular Probes; Carlsbad, Calif., US) was diluted in DMSO and applied on slides for 30 minutes at room temperature, followed by two washing steps with PBS. Slides were mounted with VECTASHIELD (Vector Laboratories,Burlingame, CA, US).

Slides were examined with epifluorescence optics. Images were acquired using the Apotome Zeiss microscope Observer Z.1 (Carl Zeiss, Jena, Germany).

### Immunoprecipitation, electrophoresis and immunoblotting

Protein lysates were prepared and analysed by a conventional Western blot (WB) assay as described elsewhere^6^. Signals from enhanced chemiluminescence reagent (ECL, Amersham, USA), used in the WB assay, were acquired by a ChemiDoc XRS + (Bio-Rad, USA) with Image Lab™ software. Antibodies: Cofilin (E-8):sc-376476, Cbl (C-15):sc-170, Actin (C4):sc-47778, Ub (P4D1):sc-8017 from Santa-Cruz Biotechnology, (Santa Cruz, CA, USA). The rat anti-LMP2A monoclonal antibodies (8C3, 14B7 and 4E11) from Ascenion GmbH (Munich, Germany), GAPDH (MA5-15738-BTIN) from ThermoFisher Scientific (USA), K48 Ub (D058-8) from MBL International (Woburn, MA, USA) K63 Ub (HWA4C4) from Enzo Life Sciences. (Farmingdale, MA, USA) Anti-Mouse IgG-HRP (#170-6516) and anti-Rabbit IgG-HRP (#170-65-15) were from Bio-Rad Laboratories (USA).

### Scratch wound assay

HEK293 cells, were seeded in a 6 well plate 24 hrs before transfection to achieve 70% confluency at the day of treatment. Cells were then transfected with 1 mkg of LMP2A, RFP-cofilin or empty vector constructs in combinations as indicated. A scratch with a P10 pipette tip was made 16 hr post-transfection. Images of scratches were recorded with a Olympus CKX 41 microscope at 4x magnification and processed using DpxView Pro [1.14.3] software at 16hr after the scratch. The remaining width at 15 positions along the scratch gap at 16 hours was normalized to the gap measured at 0 hours. The bar graph in Fig. 5C shows average width of the scratch at 0h, normalized to 1 (bars 1 and 3) with standard deviation. Bars 3 and 4 represent the ratio of the 0h value (=1) divided by the average scratch width at 16 h, resulting in a measure that reflects cell migration rate.

## Electronic supplementary material

Supplementary Information
