# Supplementary material for: Degradation of cofilin is regulated by Cbl, AIP4 and Syk resulting in increased migration of LMP2A positive nasopharyngeal carcinoma cells
Source: Sci Rep. 2017 Aug 21;7:9012. doi: 10.1038/s41598-017-09540-3 (PMC5567079; doi:10.1038/s41598-017-09540-3)
Supplement: Supplementary file 1 — Supplementary Information [file 41598_2017_9540_MOESM1_ESM.pdf]

Degradation of cofilin is regulated by Cbl, AIP4 and Syk resulting in increased migration of LMP2A positive nasopharyngeal carcinoma cells

Murat R. Gainullin[2,3], Ilya Yu. Zhukov[2,4], Xiaoying Zhou[5], Yingxi Mo[6], Lidiia Astakhova[1,7], Ingemar Ernberg[1], Liudmila Matskova[1].

[1] Department of Microbiology, Tumor and Cell Biology, Karolinska Institutet, Sweden

[2] Central Research Laboratory, Nizhniy Novgorod State Medical Academy, Nizhniy Novgorod, Minin Sq. 10/1, 603005, Russia

[3] Institute of Information Technology, Mathematics and Mechanics, Nizhniy Novgorod State University, Nizhniy Novgorod, Gagarin Av. 23, 603950, Russia

[4] Institute of Biology and Biomedicine, Nizhniy Novgorod State University, Nizhniy Novgorod, Gagarin Av. 23, 603950, Russia

[5] Medical Research Center, Guangxi Medical University, Nanning, China

[6] Department of Research, Affiliated Tumor Hospital of Guangxi Medical University, Nanning, China

[7] Institute of Food Science and Technology, Kemerovo, Russia

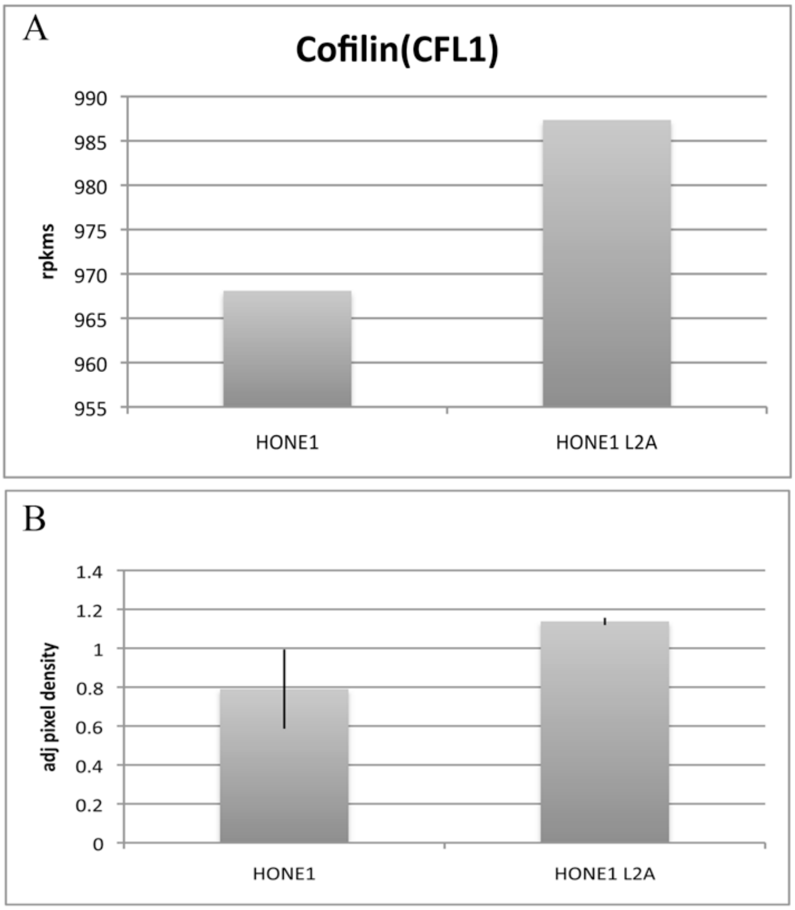

Figure S1. Cofilin gene expression is increased in the LMP2A positive HONE1 NPC cells.

A. RNAseq data (Astakhova et al, PLoS One 2016). B. RT-PCR results.

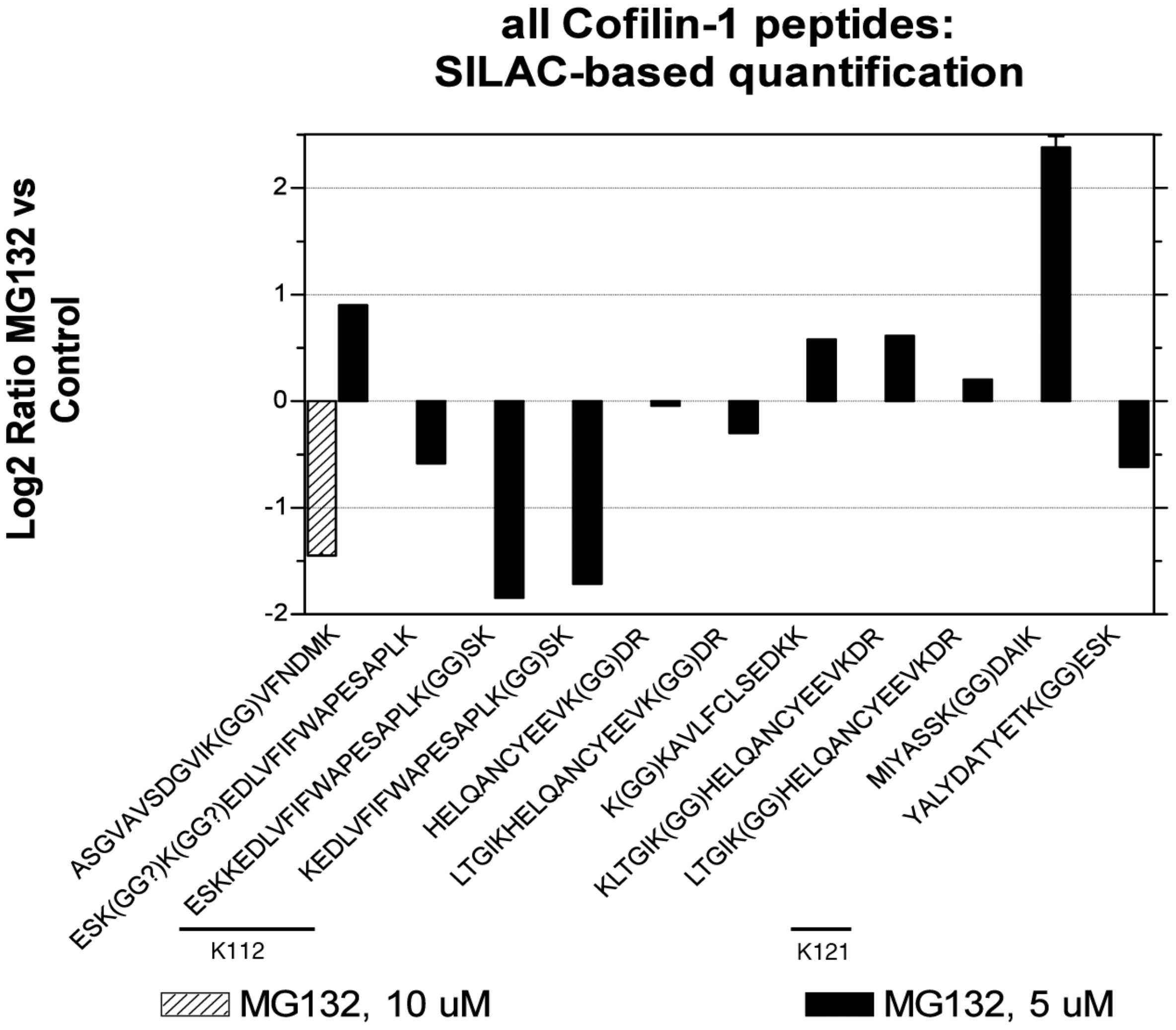

Figure S2. SILAC analysis (36) of cofilin peptide accumulation after tryptic digestion of Jurkat T cell lysates upon inhibition of proteasomal degradation with MG132 (24).

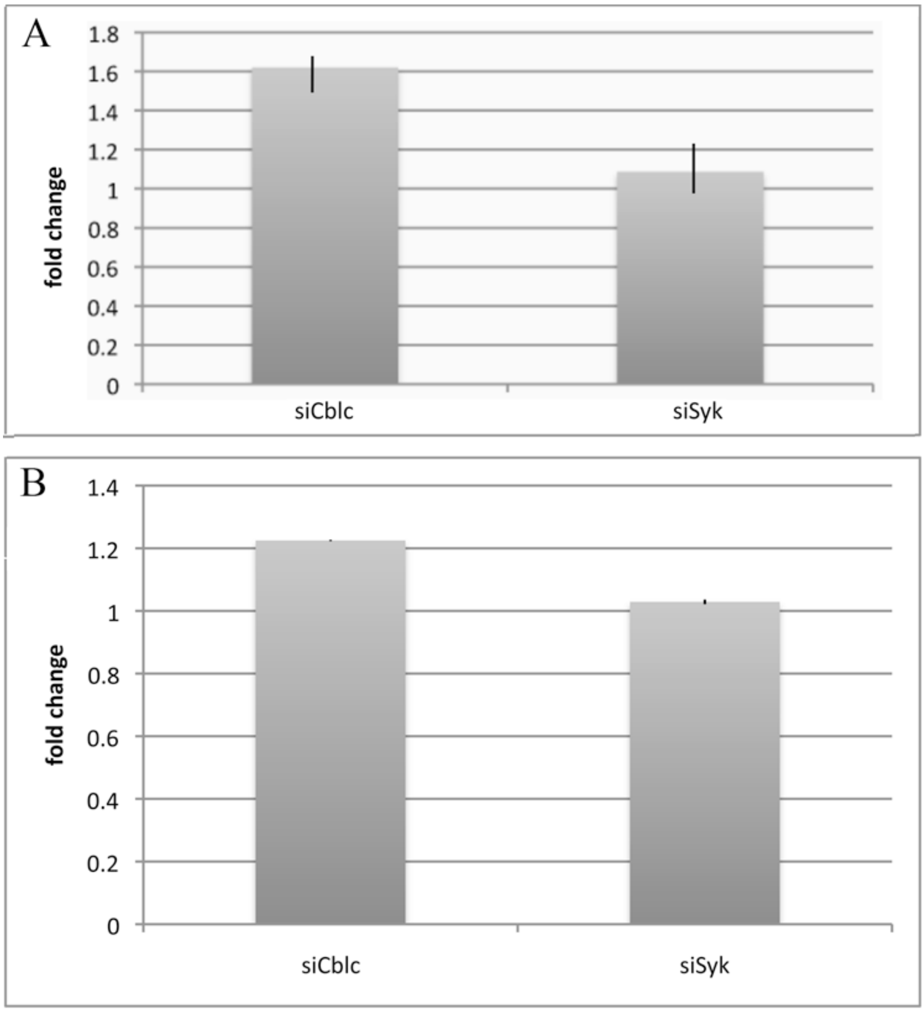

Figure S3. Down-regulation of Cbl and Syk expression results in increased cofilin expression. Western blot (A) and FACS (B) analysis of RFP-cofilin expression in CNE2 cells upon transient transfection with siRNA against either Cbl or Syk.

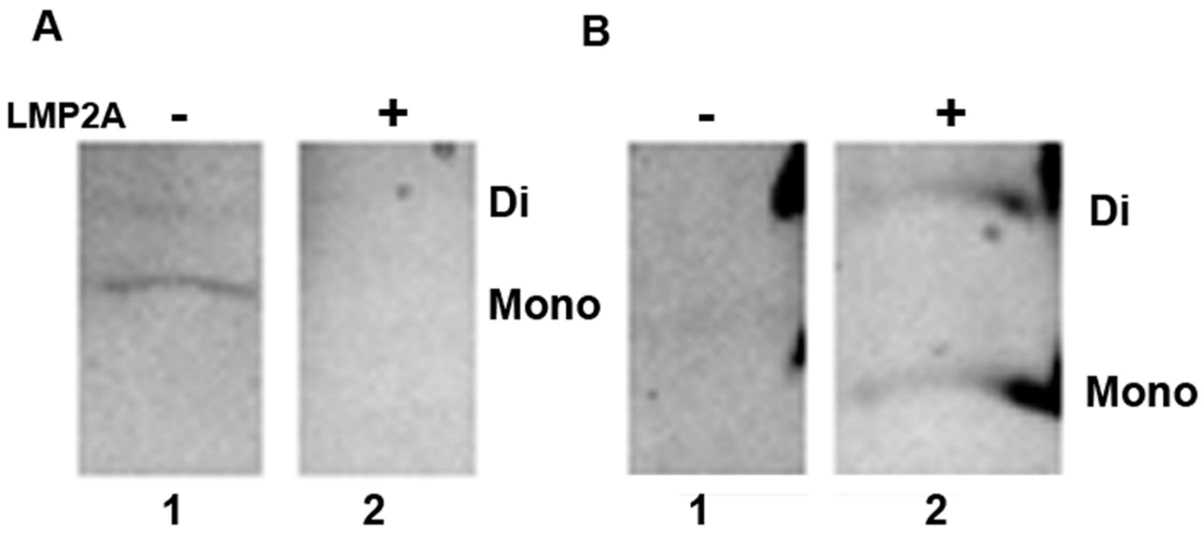

Figure S4. LMP2A expression influences sensitivity of Cofilin immunopresipitates to different DUBs. Silver stained gel of supernatants of cofilin immunoprecipitates from LMP2A positive (lanes 2) and negative (lanes 1) CNE2 cells after treatment with the K11-ubiquitin linkage specific Cezanne DUB (panel A) and the K63-ubiquitin linkage specific AMSH DUB (panel B) from a panel of DUBs, which specifically recognize different ubiquitin linkages (UbiCREST DUB Enzyme Set, K-400,Bio-techne Ltd).

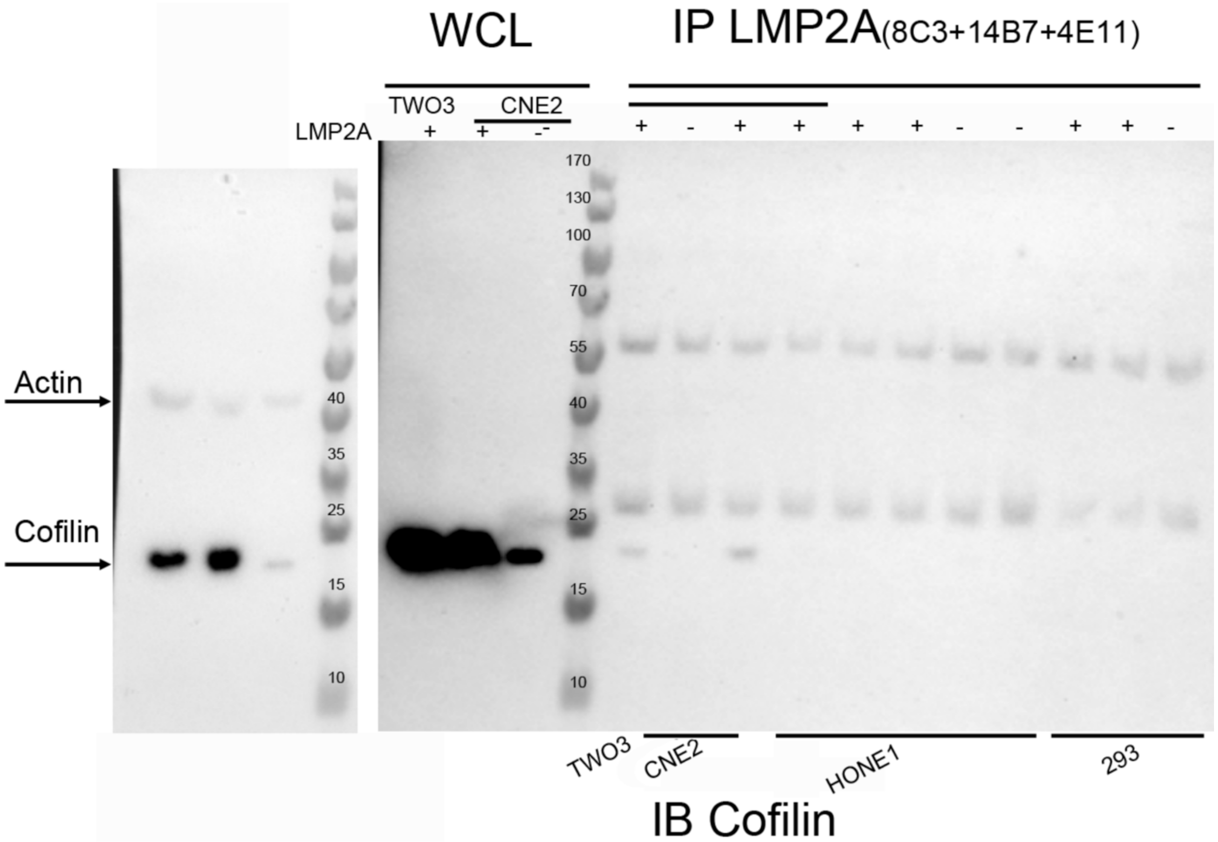

Figure S5. Full-length blot to Figure 1A. LMP2A binds cofilin and influences its stability. WB analysis of cofilin levels in wcl of the NPC cell line CNE2.

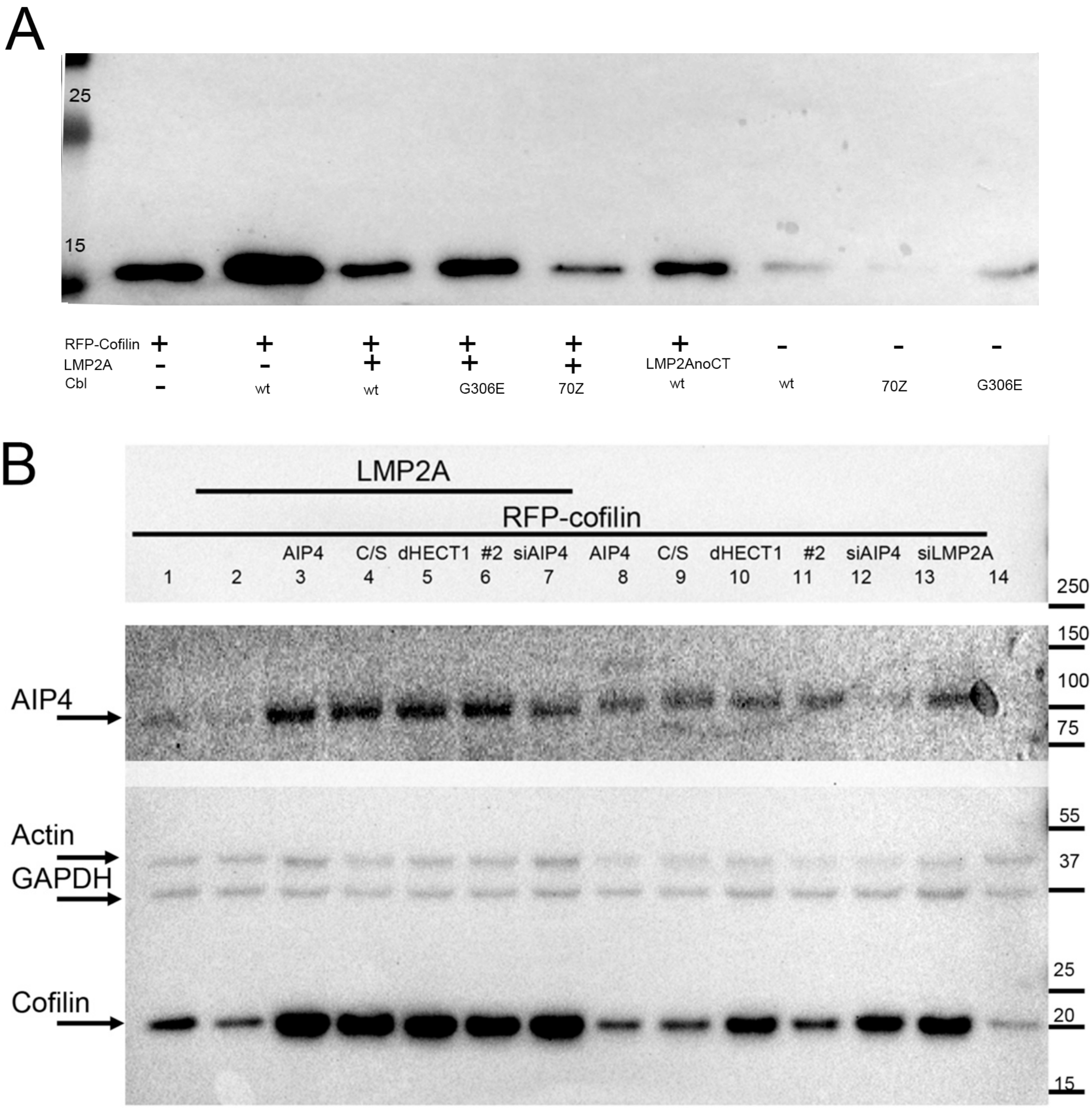
A
25
15
RFP-Cofilin + + + + + + - - -
LMP2A - - + + + LMP2AnoCT
Cbl - wt wt G306E 70Z wt wt 70Z G306E
B
LMP2A
RFP-cofilin
AIP4 C/S dHECT1 #2 siAIP4 AIP4 C/S dHECT1 #2 siAIP4 siLMP2A
1 2 3 4 5 6 7 8 9 10 11 12 13 14
250
150
100
75
AIP4
55
37
Actin
GAPDH
25
20
15
Cofilin

C

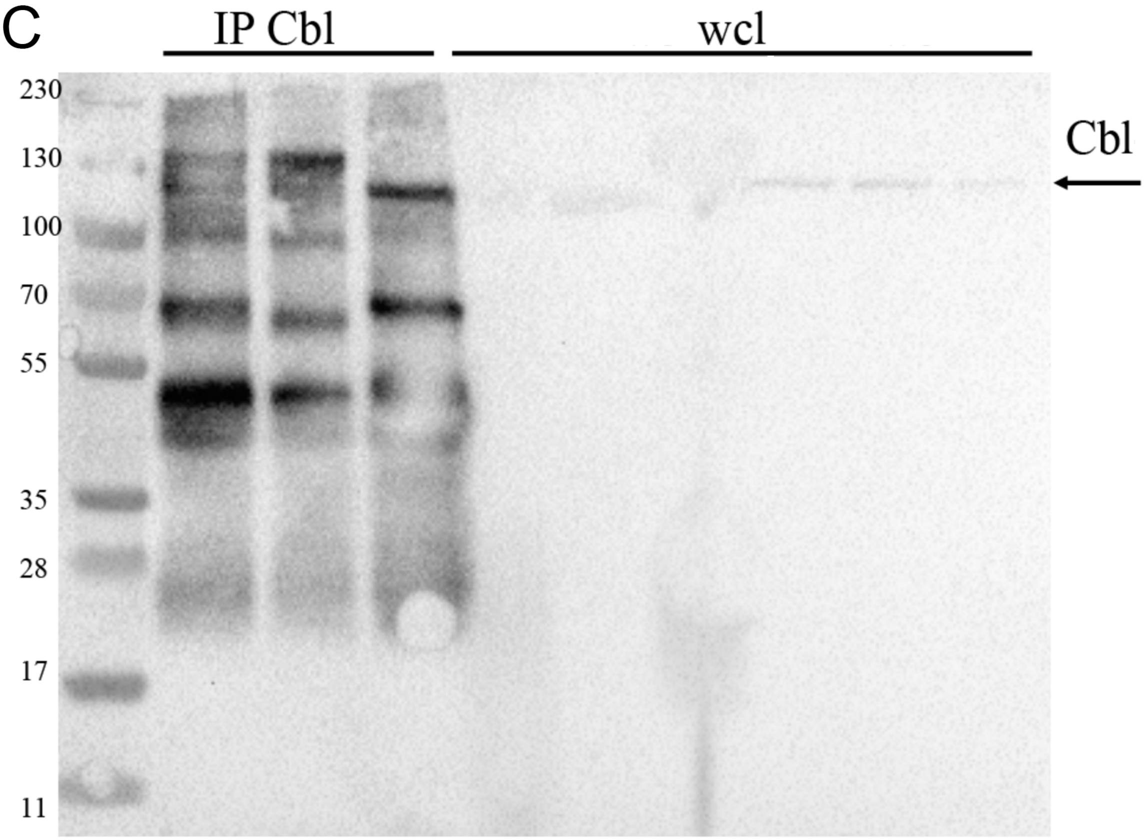

IP Cbl
wcl
230
130
100
70
55
35
28
17
11
Cbl

D

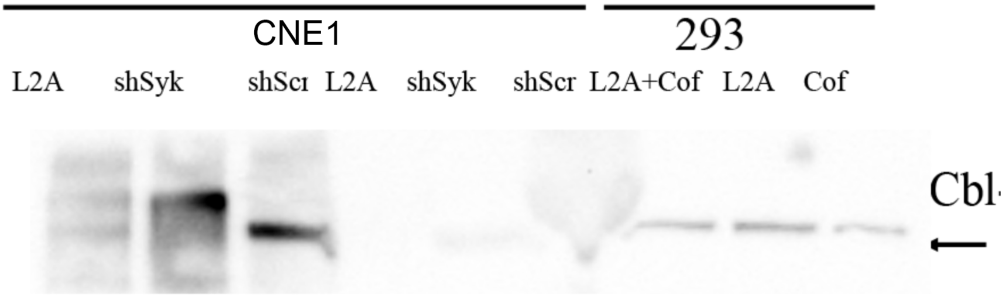

CNE1
293
L2A
shSyk
shSc
L2A
shSyk
shSc
L2A+Cof
L2A
Cof
Cbl

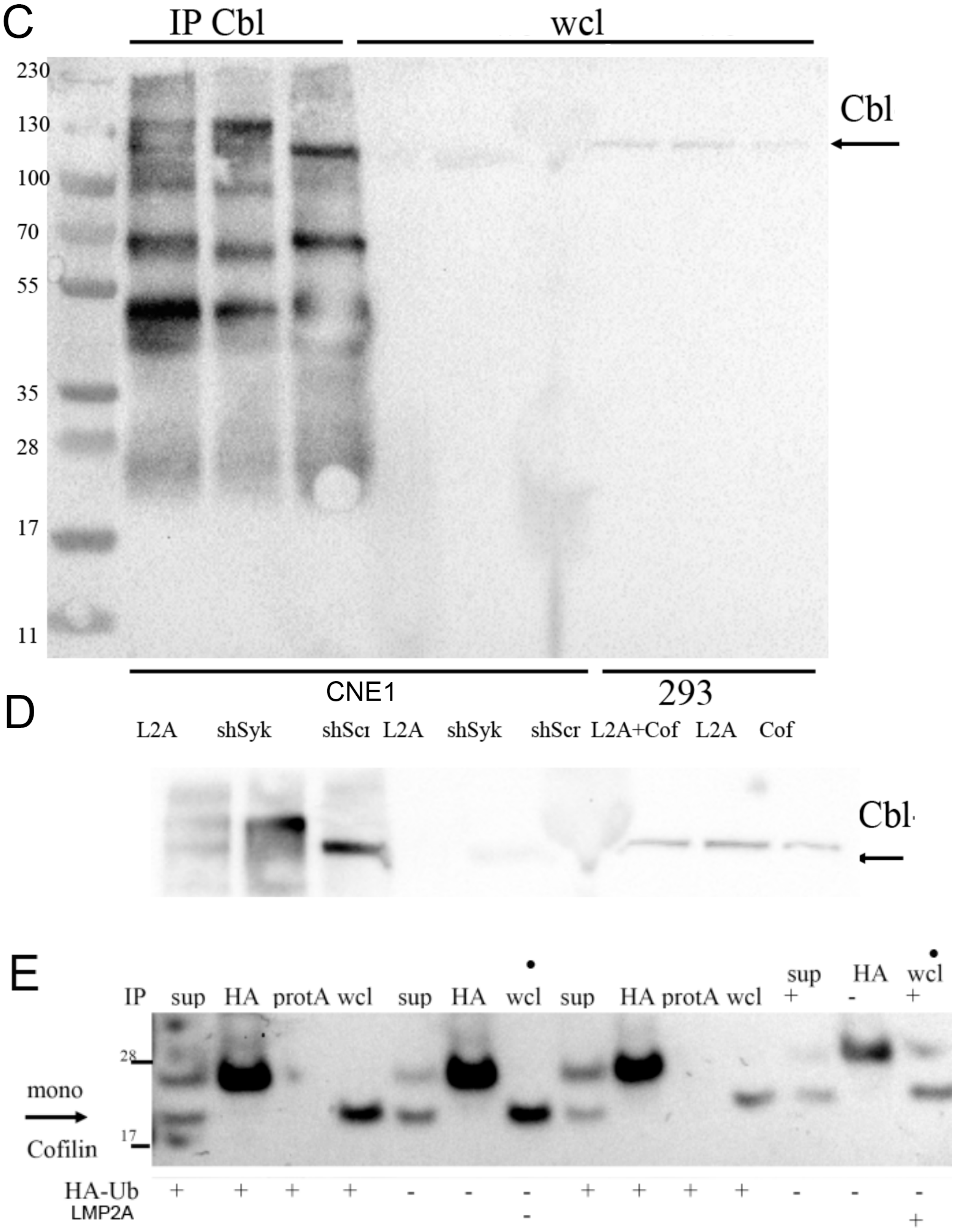
C
IP Cbl
wcl
230
130
100
70
55
35
28
17
11
Cbl
CNE1
293
D
L2A
shSyk
shScr
L2A
shSyk
shScr
L2A+Cof
L2A
Cof
Cbl
E
IP
sup
HA
protA
wcl
sup
HA
wcl
sup
HA
protA
wcl
sup
HA
wcl
28
17
mono
Cofilin
HA-Ub
LMP2A

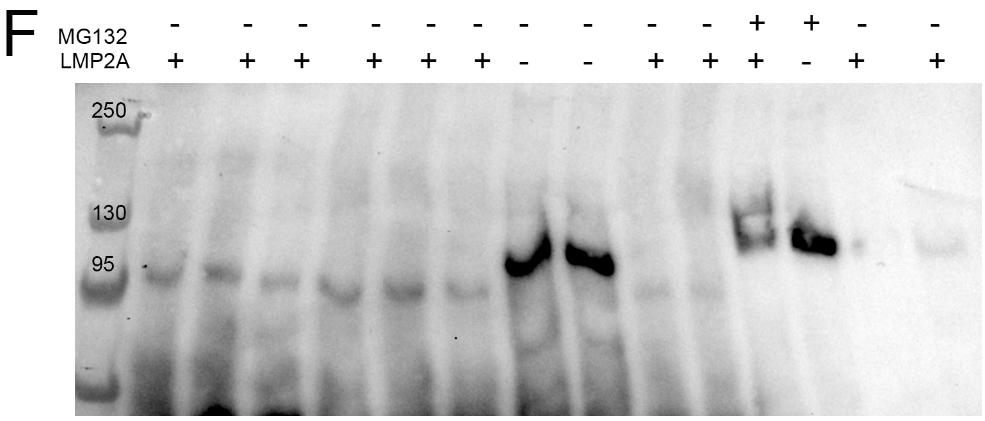

Figure S7. Full-length blots to Figure 3. Two ubiquitin E3 ligases are involved in the regulation of cofilin protein stability. WB analysis of cofilin expression in HEK293 cells upon transient expression of wt and mutant forms of Cbl (panel A) or AIP4 (panel B)

The Cbl immunoprecipitations show the appearance of post-translationally modified (larger) forms of Cbl in LMP2A or shRNAsyk expressing cells. There two different exposures of the western blot are presented on figure S7C and D. Anti-cofilin immunoblot in Panel E show that additional larger form of cofilin (27 kDa) disappears in wcl of LMP2A positive TWO3 cells (compare lanes labeled with dots). Panel F presents anti-Cbl immunoblot of LMP2A positive and negative TWO3 cells.
